# Enantioselective Toxic Effects of Prothioconazole toward *Scenedesmus obliquus*

**DOI:** 10.3390/molecules28124774

**Published:** 2023-06-15

**Authors:** Qingqing Xiang, Ying Zhou, Chengxia Tan

**Affiliations:** 1 College of Chemical Engineering, Zhejiang University of Technology, Hangzhou 310014, China; xiangqingqing163@163.com (Q.X.); tanchengxia@zjut.edu.cn (C.T.); 2 Environmental Microplastic Pollution Research Center, Zhejiang University of Technology, Hangzhou 310014, China

**Keywords:** prothioconazole, toxicity effects, *Scenedesmus obliquus*, enantioselectivity

## Abstract

Prothioconazole (PTC) is a broad-spectrum triazole fungicide with one asymmetric center and consists of two enantiomers, *R*-(−)-PTC and *S*-(+)-PTC. To address the concern of its environmental safety, the enantioselective toxic effects of PTC on *Scendesmus obliquus* (*S. obliquus*) were investigated. PTC racemates (*Rac*-PTC) and enantiomers exhibited dose-dependent acute toxicity effects against *S. obliquus* at a concentration from 1 to 10 mg·L^−1^. The 72 h-EC50 value of *Rac*-, *R*-(−)-, and *S*-(+)-PTC is 8.15, 16.53, and 7.85 mg·L^−1^, respectively. The growth ratios and photosynthetic pigment contents of the *R*-(−)-PTC treatment groups were higher than the *Rac*- and *S*-(+)-PTC treatment groups. Both catalase (CAT) activities and esterase activities were inhibited in the *Rac*- and *S*-(+)-PTC treatment groups at high concentrations of 5 and 10 mg·L^−1^, and the levels of malondialdehyde (MDA) were elevated, which exceeded the levels in algal cells for the *R*-(−)-PTC treatment groups. PTC could disrupt the cell morphology of *S. obliquus* and induce cell membrane damage, following the order of *S*-(+)-PTC ≈ *Rac*-PTC > *R*-(−)-PTC. The enantioselective toxic effects of PTC on *S. obliquus* provide essential information for its ecological risk assessment.

## 1. Introduction

Triazole fungicides are widely used to control pests and diseases in agricultural systems as the second largest fungicides [1]. About 84% of triazole fungicides are chiral, and most of them are sold and used in the form of racemates [2,3]. They may contain enantiomers, which ineffectively target organisms or are even harmful to non-target organisms [4]. Consequently, the ecological security and stereoselective bioactivities of chiral triazole fungicides should not be ignored, which play an important role in developing efficient and low-toxic green pesticides.

Prothioconazole (CAS:178928-70-6, PTC), as a highly effective broad-spectrum triazole fungicide, is extensively used in agriculture to battle diseases in crops by inhibiting the ergosterol biosynthesis of fungi [1,5]. It has become a mainstream product in fungicides, ranking third in fungicide sales and first in cereal fungicides [1,6]. The widespread use of PTC has inevitably led to residue in various environmental matrices. PTC has been detected in small agricultural streams of Norway and Germany, where the median total concentration of 76 pesticides, including PTC, was 0.18 µg·L^−1^ in Germany, and rainfall events could increase their concentration by 10 times [7,8]. 36% of Serbia soil samples have PTC residues with a concentration of 0.08 ± 0.11 μg·kg^−1^ [9]. PTC and its metabolites were also detected in air samples from Germany [10]. The residues of PTC with high concentrations (365 μg·kg^−1^) were also detected in the bee pollen of Poland [11].

**Figure 1:**
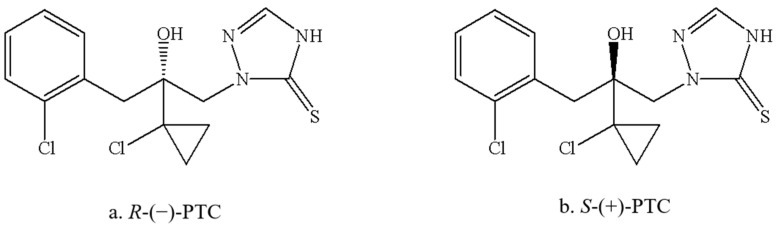
Structures of PTC enantiomers.

PTC has one asymmetric center and consists of two enantiomers, *R*-(−)-PTC and *S*-(+)-PTC (Figure 1). It showed remarkable stereoselective bioactivities. *R*-(−)-PTC was 85 to 2768 times more active than *S*-(+)-PTC and *Rac*-PTC against five target pathogens, including wheat phytoalexin, rice blast fungus, exserohilum turcicum, *Alternaria triticina*, and *Fusarium avenaceum*, with an EC_50_ ranging from 0.01 to 0.03 mg/L [12]. Similar higher antifungal activity of *R*-(−)-PTC (6-262 times) was also observed by Zhang et al. (2019) [3]. Additionally, the degradation and accumulation of PTC enantiomers are also enantioselective. The faster degradation of *R*-(−)-PTC in the soil was reported with a half-life within six days in comparison with *S*-(+)-PTC, while S-(+)-PTC preferentially accumulated in earthworms (*Eisenia fetida*) [13]. *R*-(−)-PTC was preferentially degraded in tomatoes, cucumbers, and peppers under greenhouse conditions [14]. The existing studies have focused on the activity evaluation and residual dynamics of PTC [1,3,12,14], while their toxicological effects of them were poorly understood, particularly in aquatic organisms.

PTC has a high water solubility (300 mg·L^−1^) and is stable to hydrolysis (half-lives = 17.8–34.7 d) [1,15]. It was reported to have acute toxicity on *Chlorella pyrenoidosa* (EC_50_ = 9.33 mg·L^−1^) [12], *Lemna mino L.* (EC_50_ = 1.85 mg·L^−1^) [12], *Daphnia magna* (EC_50–48 h_ = 2.82 mg·L^−1^) [16], and *Zebrafish embryos* (LC_50–96 h_ = 1.70 mg·L^−1^) [17]. PTC could induce developmental toxicity, cardiovascular effects, and oxidative damage in zebrafish [1,5,17]. It also adversely affected the growth and reproduction of *Daphnia magna* [16]. However, the enantioselective effects of PTC on aquatic organisms are very limited, and thus it is essential to further investigate their toxicological effects.

Microalgae are the dominant and primary producers in aquatic ecosystems and play a vital role in oxygen production, as well as the nitrogen and phosphorus biogeochemical cycle [18,19]. Minor disruptions of them may cause huge impacts on the entire ecosystem [18,19]. Thus, the toxicity effect of chiral PTC on microalgae merits more attention.

In this study, the toxicity effects of *Rac*-PTC and PTC enantiomers on *S. obliquus* were assessed. Different biomarkers, such as growth inhibition, photosynthetic pigments, oxidative damage, esterase activity, and membrane damage of *S. obliquus*, were investigated after PTC exposure. The responses of *S. obliquus* cells to PTC were further evaluated by flow cytometry. In addition, the interactions of PTC with *S. obliquus* were also analyzed by Fourier transform infrared (FTIR) spectroscopy. This research facilitates a better understanding of the enantioselective toxic effects of PTC on *S. obliquus* and provides data for ecological risk assessments of PTC and the appropriate use of PTC.

## 2. Results and Discussion

### 2.1. Enantioselective Growth Inhibition of PTC on S. obliquus

**Figure 2:**
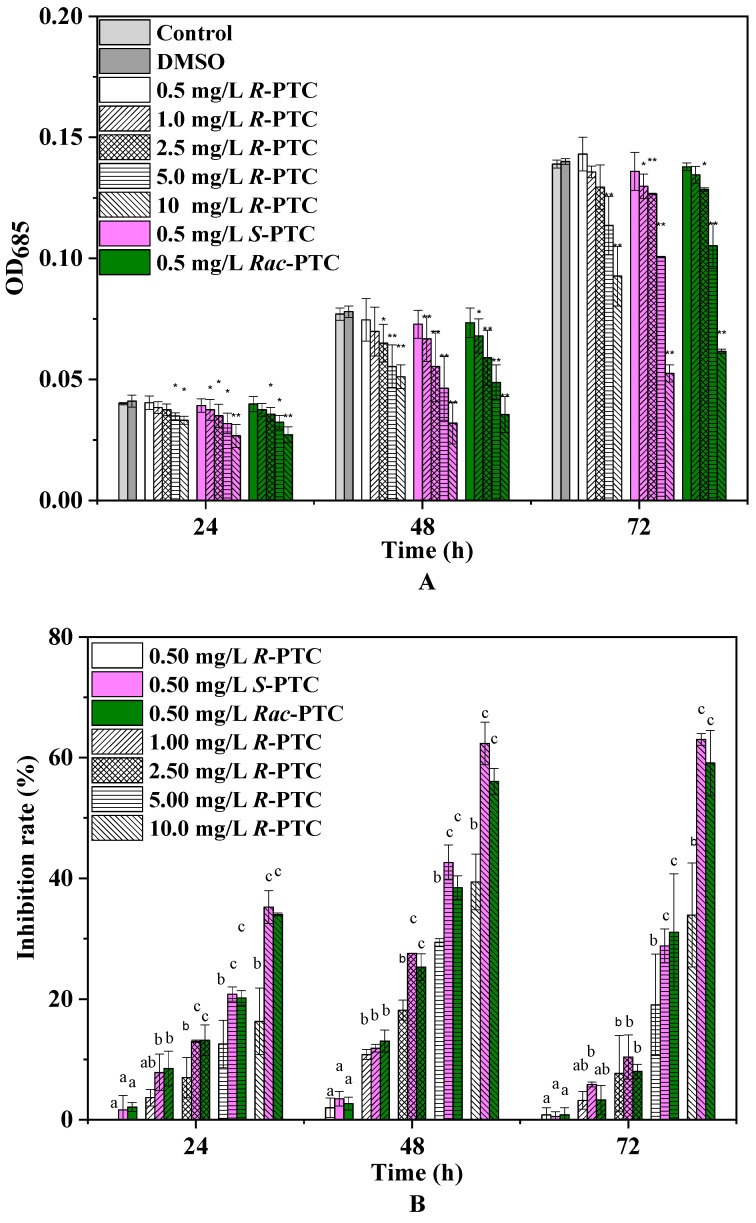
The inhibition effects of *Rac*-PTC and PTC enantiomers on *S. obliquus* growth ((**A**) the absorbance value of algae at 685 nm; (**B**) the growth inhibition rate of algae). (Lowercase letters (b and c) mean significant differences among the treatments, and ‘a’ represents no difference with the control. * means *p* < 0.05, and ** means *p* < 0.01).

The effects of *R*-PTC, *S*-PTC, and *Rac*-PTC on *S. obliquus* growth were dose-dependent, as shown in Figure 2. Compared to the control group, the growth inhibition ratio was enhanced with increasing PTC concentration in all groups except for 0.5 mg/L. Among them, the growth IR increased first and then decreased with increasing exposure time in the *R*-PTC-treated groups, with a maximum IR of 34.11%. A similar trend was observed at lower concentrations of *S*-PTC and *Rac*-PTC (≤5 mg·L^−1^), while the growth IR was enhanced with time at high concentrations (10 mg·L^−1^), with a maximum IR of 63.69% and 55.25%, respectively. These results indicated that *S. obliquus* was more sensitive to *S*-PTC and *Rac*-PTC in comparison with *R*-PTC, but the significant differences between the *S*-PTC/*Rac*-PTC and *R*-PTC treatment groups were observed at higher concentrations (≥2.5 mg·L^−1^).

To further evaluate the acute toxicity of PTC, the EC_50_ values were calculated (Table 1). The toxicity of *S*-(+)-PTC was the highest, with a 72 h-EC_50_ value of 7.85 mg·L^−1^, followed by *Rac*-PTC (EC_50_ = 8.15 mg·L^−1^). According to the acute toxicity classification criteria of green algae, the toxicity of PTC to the *S. obliquus* of PTC showed low toxicity [15]. Likewise, Zhai et al. (2018) also found that *S*-(+)-PTC showed higher acute toxicity to *Chlorella pyrenoidosa* (EC_50_ = 89.4 mg·L^−1^) and *Lemna minor L.* (EC_50_ = 0.49 mg·L^−1^) in comparison with *R*-(−)-PTC [12]. However, the toxicity of *R*-(−)-PTC and *S*-(+)-PTC on *Chlorella pyrenoidosa* was reportedly less than *Rac*-PTC (EC_50_ = 9.33 mg·L^−1^), and their acute toxicity to *Lemna minor L.* was greater than *Rac*-PTC [12]. These discrepancies might be due to the different model organisms used in the experiment. A recent study found that the acute toxicities of *S*-(+)-PTC and *Rac*-PTC to *Chlorella pyrenoidosa* were higher than *R*-(−)-PTC, with 48 h-EC_50_ values of 9.64 and 12.21 mg/L, respectively [15]. This trend is in consistent with our observation on the stereoselective toxicity of PTC toward *S. obliquus*. Although the acute toxicities of PTC racemates and enantiomers to microalgae have been reported [12,15], the toxic effects and potential toxicity mechanisms have not been explored.

### 2.2. Effects of PTC Racemates and Enantiomerson Algal Photosynthetic Pigments

**Figure 3:**
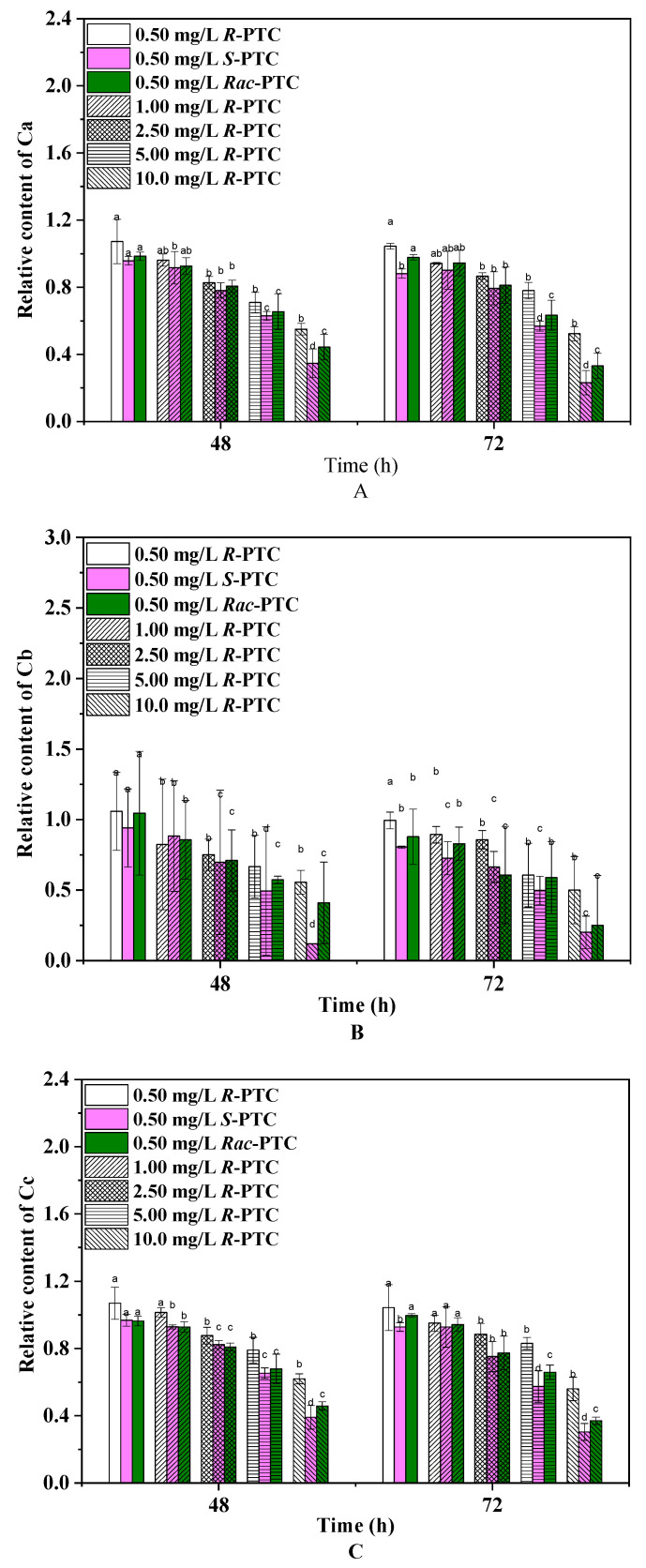
Effects of PTC racemates and enantiomers on photosynthetic pigments of *S. obliquus* ((**A**)-Ca, (**B**)-Cb, and (**C**)-Cc) (lowercase letters (b, c and d) mean significant differences among the treatments, and “a” represents no difference with the control).

Photosynthetic pigments are the basis for algae photosynthesis, and their changes can reflect algae growth [20]. Chlorophylls play a crucial role in light capture, energy transfer, and light conversion into chemical energy, while carotenoids protect the production of chlorophyll and absorption of light energy, playing an important role in the stress signaling pathway [19,20]. Thus, the effects of the racemates and enantiomers of PTC on pigments were investigated. As shown in Figure 3, the relative contents of Ca, Cb, and Cc showed a dose-dependent reduction trend, except for 0.5 mg·L^−1^. Notably, *R*-(−)-PTC could increase the contents of photosynthetic pigments at 0.5 mg/L as compared with the control. Likewise, an increase of Ca and Cc was also observed in the 0.5 mg·L^−1^ *Rac*-PTC treatment group. It might be due to the fact that algae possibly took advantage of a supply of carbon and nitrogen sources coming from the *Rac*-PTC and *R*-(−)-PTC at low concentrations [21,22]. The diminution of the algae photosynthetic pigment content under high concentrations of PTC (>0.5 mg/L) was probably due to the peroxidative breakdown of pigments and membrane lipids by ROS [20,23,24]. A similar reduction trend was observed by Nong et al. (2021) when they studied the impacts of three common azole fungicides (myclobutanil, propiconazole, and tebuconazole) on chlorophyll synthesis in *Chlorella pyrenoidosa* [23]. In addition, significant enantioselective inhibition effects of PTC on Ca and Cc were observed at high concentrations (≥5 mg·L^−1^) after 72 h of exposure, with the order of *S*-(+)-PTC > *Rac*-PTC > *R*-(−)-PTC. Regarding Cb, the greatest remarkable decrease in the *S*-(−)-PTC-treated groups were observed in comparison with the *R*-(+)-PTC and *Rac*-PTC treatment groups after 72 h of exposure. These findings meant that PTC could affect algae growth by interfering with algae photosynthesis.

### 2.3. Effects of PTC on Intracellular Oxidative Stress and Esterase Activities

**Figure 4:**
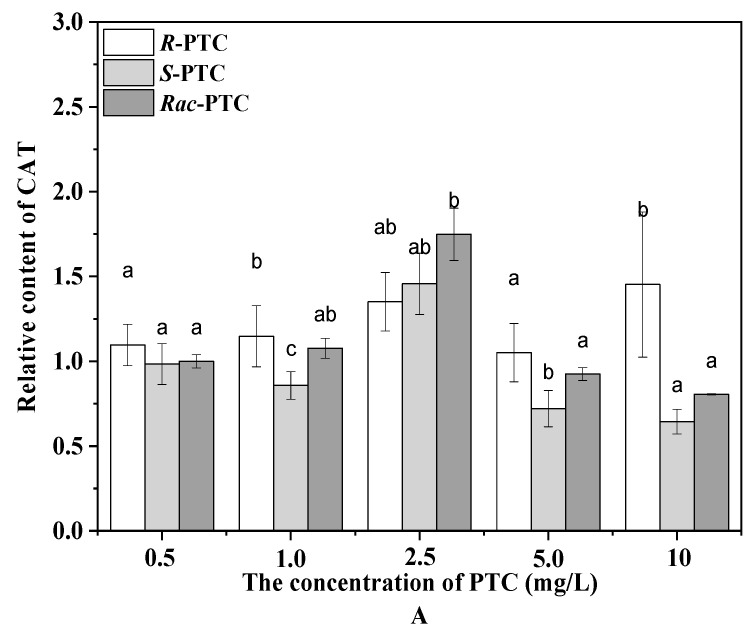

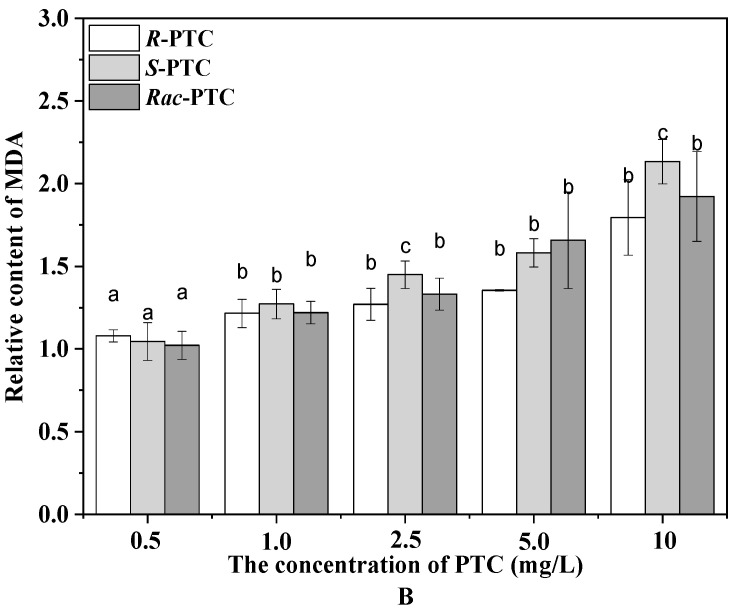
Relative contents of CAT (**A**) and MDA (**B**) in *S. obliquus* after 72 h exposure to PTC (lowercase letters (b and c) mean significant differences among the treatments, and “a” represents no difference with the control).

Algae can produce ROS during different metabolic pathways in mitochondria, chloroplasts, and peroxisomes under environmental stress [20,24]. To maintain the steady-state levels of ROS in the cell, an antioxidant enzyme participates in antioxidant protection processes. The CAT catalyzes the degradation or reduction of H_2_O_2_ to water and oxygen molecules [21,24]. In this study, the content of the CAT was increased first and then decreased with the increase of the PTC concentration upon treatment with *Rac*-PTC and *S*-(+)-PTC, while the CAT activity was enhanced with the increased concentration of *R*-(−)-PTC, except for 5 mg·L^−1^ (Figure 4A). The initial increase of the CAT induced by *Rac*-PTC and *S*-(+)-PTC might be due to hormesis [20,21], whereas the decrease in the CAT might be attributed to the overproduction of ROS, because it could destroy the intrinsic antioxidant defense of cells, resulting in lethal damage to the cells [21,23]. These data indicated that *Rac*-PTC and *S*-PTC could destroy the antioxidant system of algal cells at high concentrations (≥5 mg·L^−1^), which was further confirmed by the enhanced MDA content (Figure 4B). MDA is a product of lipid peroxidation and is generally considered a biomarker of oxidative stress [21,24]. The MDA content increased significantly with the increasing concentrations of PTC with respect to the control, except for 0.5 mg/L of PTC (Figure 4B). Compared with *R*-(−)-PTC, the MDA content was higher in the *Rac*-PTC and *S*-(+)-PTC treatment groups. These discoveries revealed that PTC could induce enantioselective oxidative damage to *S. obliquus* at high concentrations, with the toxicity order of *S*-(+)-PTC ≈ *Rac*-PTC > *R*-(−)-PTC.

**Figure 5:**
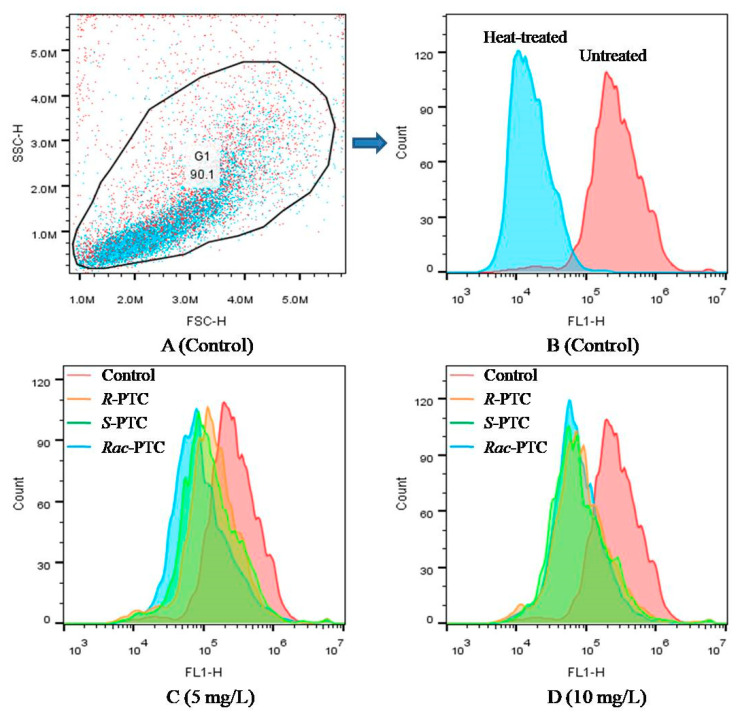
Effect of PTC racemate and its enantiomers on the esterase activity of *S. obliquus* (25 µM FDA, FL1).

Esterase activity is a critical biochemical parameter used to assess phytoplankton metabolic activity [25]. Considering the difference in CAT activities among the *R*-(−)-PTC, *S*-(+)-PTC, and *S*-(+)-PTC treatment groups at high concentrations (≥5 mg·L^−1^), the esterase activity was further analyzed by FDA staining in combination with flow cytometry to better understand the toxicity mechanism of PTC on algae. FDA is a nonfluorescent lipophilic molecule that can freely cross the plasma membrane and produce green fluorescence after hydrolysis by nonspecific esterases within living cells [25,26]. Therefore, the change of fluoresce in fluorescence intensity can reflect esterase activity. As shown in Figure 5, the mean fluorescence intensity (MFT) of FL1 decreased after treatment with 5 and 10 mg·L^−1^ of PTC with respect to the negative control, especially at 10 mg·L^−1^. The decrease in MFT might be due to the quenching of esterase activity or the destruction of cell membranes, reducing the absorption of the dye [25,26]. The effects of PTC on esterase activity showed enantioselectivity. *R*-(−)-PTC showed the greatest MFT in contrast with *S*-PTC and *Rac*-PTC. This result, together with the above oxidative stress effect, indicated that the toxicity effect of *S*-PTC and *Rac*-PTC on *S. obliquus* was higher than *R*-PTC.

### 2.4. FTIR Analysis to Evaluate the Interaction between PTC and Microalgae

The first self-protection barrier of algae consists of cell walls and cell membranes [27,28,29]. They are composed of cellulose, polysaccharides, phospholipids bilayer, proteins, carbohydrates, and other substances [27,28]. These biomacromolecules play an important role in the stability of the cell surface structure and cell function, which are vital signs in toxic evaluation [27,28]. They have spectral characteristic bands, such as lipids (1726 cm^−1^, C=O), proteins (N-H 3300 cm^−1^, Amide I 1639 cm^−1^, and Amide II 1541 cm^−1^), and carbohydrates (1200–800 cm^−1^, C-O, C-H) [28,29]. The variations in peak intensities can reflect the changes in the biochemical composition of the algae surface upon PTC treatment [28]. Thus, the interaction and potential impacts of PTC on microalgae were analyzed by FTIR.

**Figure 6:**
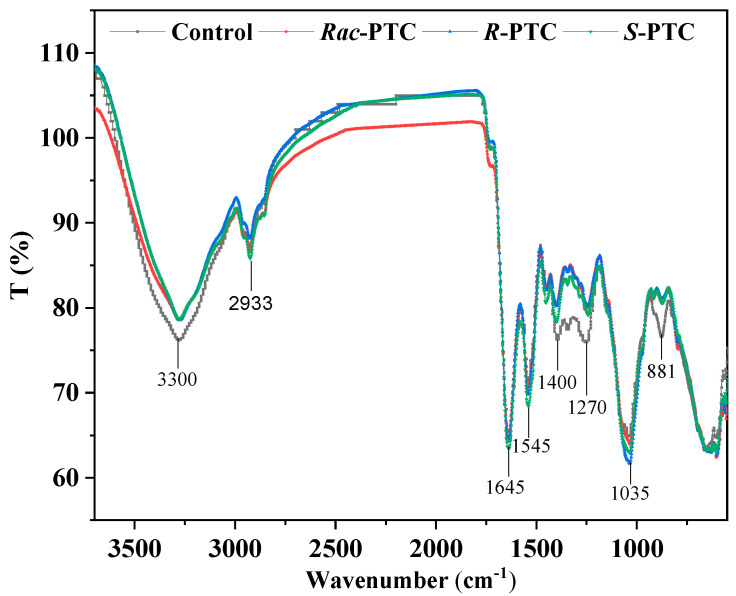
Infrared spectra of *S. obliquus* cells after treatment with 5 mg·L^−1^ of PTC.

Compared with the control, the intensity of the protein-related peak at 3300 cm^−1^ (N–H, Amide II) and the carboxylate group (COO-) of protein at 1450 to 1300 cm^−1^ were decreased when *S. obliquus* co-existed with 5 mg·L^−1^ *Rac*-PTC and its enantiomers for 72 h (Figure 6). Likewise, the protein-related band at 1650 cm^−1^ (N–H, C-O, Amide I) [29] was shrunk in the *Rac*-PTC and *R*-PTC treatment groups. These decreases were accredited to the changes in the protein structures on the algae surface. Apart from protein-related bands, the intensity of the phosphorylated molecules correlation band (P=O, 1270 cm^−1^) was also decreased after the treatment of *Rac*-PTC and its enantiomers, which indicates that PTC could induce cell membrane damage [30]. In contrast, the intensity of the polysaccharide/carbohydrate-related peak (1035 cm^−1^) was slightly increased with a blue shift trend after the PTC treatments. In addition, it should be noted that one of the polysaccharide/carbohydrate-related peaks (C-H, 881 cm^−1^) [31] decreased and even disappeared after treatment with 5 mg·L^−1^ of PTC and its enantiomers. These findings indicated that the interaction between PTC and *S. obliquus* could interfere with the composition of biomacromolecules on the algae surface, thus affecting the cell surface structure. Further molecular studies may be useful for exploring the interaction mechanisms between microalgae and PTC, as well as the associated metabolic changes.

### 2.5. Effects of PTC on Cell Morphology and Membrane Permeability of S. obliquus

**Figure 7:**
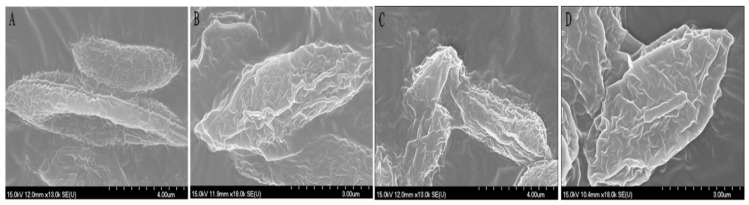
Effects of PTC racemate and its enantiomers on the morphology of *S. obliquus.* (**A**) Control; (**B**) 10 mg·L^−1^ *R*-PTC; (**C**) 10 mg·L^−1^ *S*-PTC; (**D**) 10 mg·L^−1^ *Rac*-PTC.

The variations of biomacromolecules on the algae surface may be related to the damage to the cell surface structure [29,30,31]. The interactions between PTC and microalgae were investigated by SEM. As shown in Figure 7, the algal cells were deformed and wrinkled after the treatment with 10 mg·L^−1^ of PTC in comparison with the control. These changes may increase the membrane permeability and cause irreversible lesions on the cell membrane or cell wall [32].

**Figure 8:**
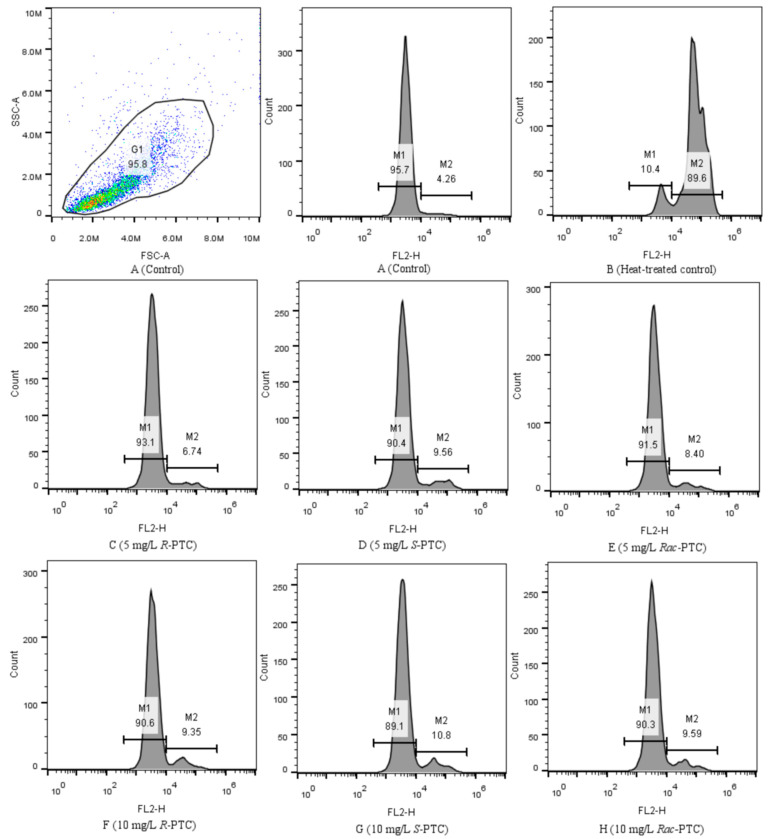
Flow cytometric histograms of *S. obliquus* stained with PI (10 µM, FL2).

To examine the damage of the cell membrane induced by the PTC, the algal cells were stained using the PI method. PI cannot pass through the intact cell membranes of live cells, but it can cross membrane-damaged cells and combine with nucleic acids, producing red fluoresce [26,33]. Normally, the healthy algal cells (the negative control, Figure 8A) fluoresced in a region of lower fluorescence intensity (M1), while dead cells (the positive control, Figure 8B) were mainly distributed in the M2 region [33]. Compared with the negative control, the dead cells (M2) increased after the treatment with 5 and 10 mg·L^−1^ of PTC, especially in the 10 mg·L^−1^ treatment groups (Figure 8). This phenomenon meant that PTC would destruct the cell membrane of *S. obliquus*, reflecting the damage to the protoplasm and phospholipid bilayers [32]. It might be a reason for the variation of biomacromolecules on the algal surface. In addition, the impacts of PTC on cell membranes showed enantioselectivity, with the order of *S*-PTC > *Rac*-PTC > *R*-PTC (Figure 8). This finding was in accord with the above result, indicating that *Rac*-PTC and *S*-PTC more easily induced membrane damage to *S. obliquus*, leading to the disorder of cell function and thus affecting algae growth.

The increase in membrane permeability may have affected the transport of nutrients in *S. obliquus* and enhanced the possibility of PTC permeating the cell membrane [21]. PTC could be rapidly converted to the highly toxic metabolite prothioconazole-desthio when it was absorbed by microalgae (*Chlorella pyrenoidosa*), particularly in the *S*-PTC treatment group [15]. It may also be a reason for the greater toxicity of *S*-PTC and *Rac*-PTC to *S. obliquus*. Given the toxicity effects of PTC on *S. obliquus*, further studies on its biodegradation and metabolism are warranted in the risk assessment and safe use of PTC [34]. Apart from the toxicity data of microalgae, microbial toxicity tests can also provide valuable data for the risk assessment of PTC. These may include respiration inhibition tests, tests for the inhibition of nitrification, luminescent bacteria tests, and growth inhibition tests [35].

## 3. Materials and Methods

### 3.1. Chemicals and Reagents

**Figure 9:**
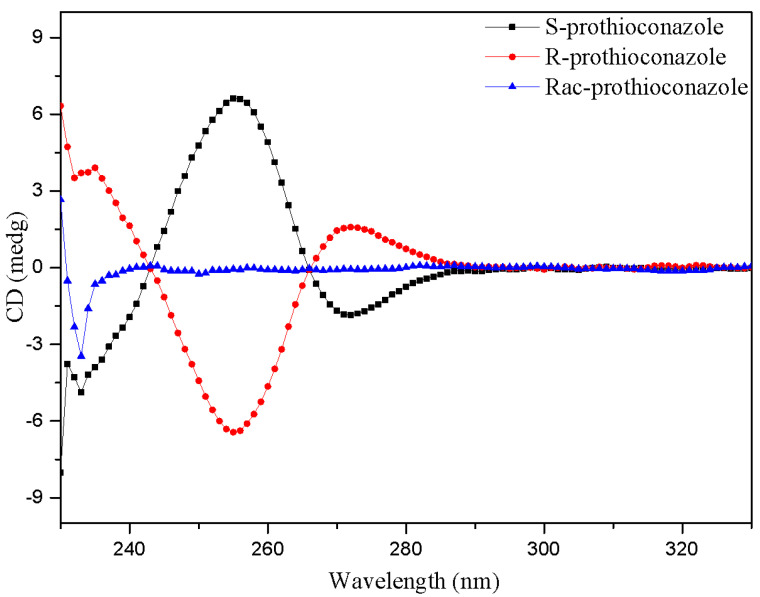
The CD spectra of PTC and its enantiomers.

PTC (purity ≥ 98%) and its enantiomers (purity ≥ 99.39%) were provided by Dr. Chengrong Ding from Zhejiang University of Technology (Hangzhou, China) and Dr. Zhaoxian Zhang from Nanjing Agricultural University (Nanjing, China), respectively. Their absolute configurations were confirmed by circular dichroism (CD) spectra (Figure 9). PTC stock solutions were prepared in DMSO (purity ≥ 99.9%, Aladdin, Shanghai, China) and stored at 4 °C. The chemicals and solvents used in this study were at least of analytical grade except for special instructions. Exposure solutions were prepared by diluting the stock solution in a BG-11 medium to achieve working solutions, with a final DMSO concentration of 0.1% (*v*/*v*) in all treatment groups.

### 3.2. Algal Growth Inhibition Test

*S. obliquus* was purchased from the Institute of Hydrobiology, Chinese Academy of Sciences, and cultured in a BG-11 medium at 24 ± 1 °C in an incubator under illumination at 4500 lx with daily cycles of 14:10 h light: dark cycle. An algal growth inhibition test was conducted according to the updated OECD guideline 201 [36]. Exponential growth phase algal cells were cultivated in 150 mL of a culture medium with a series of *Rac*-PTC or PTC enantiomer dilution concentrations (0, 0.5, 1, 2.5, 5, and 10 mg·L^−1^) in 250 mL Erlenmeyer flasks with an initial cell density of 4.33 × 10^5^ cells·mL^−1^ (OD_685_ = 0.018) for 72 h. To ensure optimum growth, the flasks were shaken five times, and their position in the incubator was randomly changed per day. Algal cell density was monitored at 685 nm by UV spectrophotometer (UNICO 2802 S, Franksville, WI, USA) and counted with a hemocytometer under an optical microscope (Nikon, Tokyo, Japan) per 24 h. Three replicates were performed for each treatment. The regression equation for the relationship between cell density (y × 1.0 × 10^5^ cells·mL^−1^) and absorption at 685 nm (x) was calculated as y = 90.16x + 2.71 (*p* < 0.01, R^2^ = 0.98).

The effective concentrations resulting in 50% inhibition (EC_50_) were calculated by a probit equation based on the growth inhibition ratios induced by PTC. The algal growth inhibition ratio (IR) was calculated using the following equation:

$${IR}_{i}\left(\%\right)=\frac{C_{ci}-C_{ti}}{C_{ci}}\times 100\%$$

where IR_i_ is the growth inhibition rate at time i; C_ci_ is the cell density of the control at time i; and C_ti_ is the cell density of the treated group at time i.

### 3.3. Determination of Photosynthetic Pigments

Algal cells were collected by centrifugation (8000 rpm, 10 min) after 48 h or 72 h of exposure. To remove the extra PTC, the concentrated algal cells were washed twice with a phosphate buffer (0.01 M, pH = 7.4), and the pigments were then extracted by adding ethanol (100%, *v*/*v*) overnight [23]. The supernatant was collected by centrifugation and analyzed by a UNICO 2802 S spectrophotometer at 663, 645, and 440 nm following the reported protocols [21,23]. The data were expressed as the relative content of the control.

The contents (mg·L^−1^) of chlorophyll a (Ca), chlorophyll b (Cb), and carotenoids (Cc) were calculated as follows:

Ca = (12.7 × OD_663_ − 2.69 × OD_645_) × V1 ÷ V2,

Cb = (20.13 × OD_645_ − 5.03 × OD_663_) × V1 ÷ V2,

Cc = (1000 × OD_440_ − 3.27 × Ca − 104 × Cb)/229 × V1 ÷ V2

where V1 (mL) and V2 (mL) are the volume of the pigment extract and microalgae solution, respectively.

### 3.4. CAT and MDA Analysis

Algal cells were collected by centrifugation (8000 rpm, 10 min) and resuspended with PBS (0.01 M, pH = 7.4). The algal cells were washed twice with PBS and then homogenized by sonication in an ice bath for 5 min. The homogenate was centrifuged, and the supernatant was collected for further analysis. Proteins were detected using the Coomassie bright blue G-250 method [37]. The molecular indicator of lipid peroxidation, malondialdehyde (MDA), was evaluated using a microscale MDA assay kit (Nanjing Jiancheng Bioengineering Institute, Nanjing, China) according to the manufacturer’s protocol. The catalase (CAT) activity was measured using a CAT assay kit (Nanjing Jiancheng Bioengineering Institute, China). All biomarker concentrations were normalized to their protein content separately.

### 3.5. Esterase Activity and Cell Viability Analysis

Membrane permeability, esterase activity, and cell viability of *S. obliquus* were analyzed by flow cytometry (EasyCell, Wellgrow, Shanghai, China), and the data were analyzed by Flowjo_V10 software. The esterase activity was determined by the fluorescein diacetate (FDA) staining method [25,26]. After 72 h of exposure, algae cells were collected by centrifugation (5000 rpm, 10 min), washed with PBS (0.01 M, pH = 7.4), and then stained with FDA at final concentrations of 25 µM and incubated in the dark for 20 min. Esterase activity was determined by FL1 (500–560 nm band-pass filter, excitation at 488 nm blue laser) after staining with FDA.

Cell viability was analyzed by propidium iodide (PI) staining according to the reported protocols [26,33]. After 72 h exposure to PTC, algae cells were collected by centrifugation (5000 rpm, 10 min), washed with PBS (0.01 M, pH = 7.4), and then stained with PI at final concentrations of 10 µM. The fluorescent emission was collected in the FL2 channel by flow cytometer.

### 3.6. FTIR Characterization of the Interactions between PTC and Algae

FTIR analysis was conducted to assess the changes of biomacromolecules on the algae surface after PTC exposure, which can be used to evaluate the degree of cell damage [38]. Algal cells were prepared by an identical procedure for FTIR analysis in the presence or absence of 5 mg·L^−1^ PTC. In brief, algal cells were harvested by centrifugation for 10 min at 8000 rpm after 72 h of exposure. The cells were then washed twice with PBS (0.01 M, pH = 7.4) and naturally dried for 1 d at 25 °C. The dried cells were analyzed by ATR-FTIR (Nicolet 6700, Thermo, Waltham, MA, USA). A spectral range from 4000 to 500 cm^−1^ was collected with an accumulation of 10 scans and a resolution of 4 cm^−1^.

### 3.7. Cell Morphology Analysis

The cell morphology of *S. obliquus* was analyzed by scanning electron microscopy (SEM) (Hitachi S-4700 (II), Tokyo, Japan) after 72 h exposure to PTC. Algal cells were collected by centrifugation (8000 rpm, 10 min) and then washed twice with PBS (0.01 M, pH = 7.4). The collected algal cells were fixed with 2.5% glutaraldehyde overnight at 4 °C and then dehydrated in gradient concentrations of ethanol. The dried samples were sputtered with gold layers and analyzed by SEM.

### 3.8. Statistical Analysis

Data were expressed as the mean ± standard deviation. The one-way analysis of variance (ANOVA) was performed by SPSS IBM Statistics software 20.0. The post-multiple comparisons were performed by Dunnett’s *t*-test. *p* < 0.05 was considered as the statistical significance and was marked with “*” or lowercase letters “b, c, and d”.

## 4. Conclusions

This study investigated the stereoselective toxicity of PTC to *S. obliquus*. PTC racemates and enantiomers have acute toxicities to the concentration-dependent inhibition effects on algal growth and photosynthesis toward *S. obliquus* at 1–10 mg·L^−1^, with the order of *Rac*-PTC ≈ *S*-PTC > *R*-PTC. The inhibition of algae growth by PTC was contributed partly by the interference with the synthesis of photosynthetic pigments. PTC could induce oxidative damage to *S. obliquus* at high concentrations (≥5 mg·L^−1^). It caused an imbalance between the antioxidant system and oxidative stress and impaired the cell morphology and membrane permeability of *S. obliquus*, especially the in *Rac*-PTC and *S*-(+)-PTC treatment groups. Additionally, PTC could interfere with the composition of biomacromolecules on the algae surface. These results revealed the stereoselective toxicities of PTC to *S. obliquus* and that the ecological risks of PTC should be assessed at the stereoselective level to reduce the hidden dangers of PTC to the environment.

## Figures and Tables

**Table 1:** The EC_50_ values of PTC racemates and enantiomers on *S. obliquus* growth.

	*R*-(−)-PTC		*S*-(+)-PTC		*Rac*-PTC	
	EC_50_ (mg·L^−1^)	R^2^	EC_50_ (mg·L^−1^)	R^2^	EC_50_ (mg·L^−1^)	R^2^
24	49.54 (25.22–211.97)	0.76	19.29 (14.03–30.75)	0.95	21.83 (15.28–37.35)	0.92
48	14.54 (11.54–19.70)	0.85	6.29 (5.58–7.20)	0.97	7.62 (6.62–8.99)	0.93
72	16.53 (11.44–32.42)	0.87	7.85 (6.99–8.98)	0.95	8.15 (6.91–10.05)	0.93

## Data Availability

Data are available from the corresponding author upon request.

## References

[1] Tian S., Teng M., Meng Z., Yan S., Jia M., Li R., Liu L., Yan J., Zhou Z., Zhu W. (2019). Toxicity effects in zebrafish embryos (*Danio rerio*) induced by prothioconazole. Environ. Pollut..

[2] Zhang Z., Du G., Gao B., Hu K., Kaziem A.E., Li L., Wang M. (2019). Stereoselective endocrine-disrupting effects of the chiral triazole fungicide prothioconazole and its chiral metabolite. Environ. Pollut..

[3] Zhang Z., Gao B., He Z., Li L., Zhang Q., Kaziem A.E., Wang M. (2019). Stereoselective bioactivity of the chiral triazole fungicide prothioconazole and its metabolite. Pestic. Biochem. Physiol..

[4] Liu R., Deng Y., Zhang W., Zhang L., Wang Z., Li B., Zhou Z. (2019). Enantioselective mechanism of toxic effects of triticonazole against *Chlorella pyrenoidosa*. Ecotoxicol. Environ. Saf..

[5] Shen J., Liu P., Sun Y., Xu X., Guo L., Rao Q., Wu H. (2020). Embryonic exposure to prothioconazole induces oxidative stress and apoptosis in zebrafish *(Danio rerio*) early life stage. Sci. Total Environ..

[6] Xie Y., Jiang H., Chang J., Wang Y., Li J., Wang H. (2019). Gonadal disruption after single dose exposure of prothioconazole and prothioconazole-desthio in male lizards (*Eremias argus*). Environ. Pollut..

[7] Stenrød M. (2015). Long-term trends of pesticides in Norwegian agricultural streams and potential future challenges in northern climate. Acta Agric. Scand. Sect. B-Soil Plant Sci..

[8] Halbach K., Möder M., Schrader S., Liebmann L., Schäfer R., Schneeweiss A., Schreiner V., Vormeier P., Weisner O., Liess M. (2021). Small streams–large concentrations? Pesticide monitoring in small agricultural streams in Germany during dry weather and rainfall. Water Res..

[9] Pap S.M., Popovic B., Stojic N., Danojevic D., Pucarevi M., Cervenski J., Speranda M. (2023). The environmental issue of pesticide residues in agricultural soils in Serbia. Int. J. Environ. Sci. Technol..

[10] Kruse-Plass M., Hofmann F., Wosniok W., Schlechtriemen U., Kohlschuetter N. (2021). Pesticides and pesticide-related products in ambient air in Germany. Environ. Sci. Eur..

[11] Roszko M., Karninska M., Szymczyk K., Jedrzejczak R. (2017). Levels of selected persistent organic pollutants (PCB, PBDE) and pesticides in honey bee pollen sampled in Poland. PLoS ONE.

[12] Zhai W., Zhang L., Cui J., Wei Y., Wang P., Liu D., Zhou Z. (2019). The biological activities of prothioconazole enantiomers and their toxicity assessment on aquatic organisms. Chirality.

[13] Wang X., Liu Y., Xue M., Wang Z., Yu J., Guo X. (2019). Enantioselective degradation of chiral fungicides triticonazole and prothioconazole in soils and their enantioselective accumulation in earthworms *Eisenia fetida*. Ecotoxicol. Environ. Saf..

[14] Jiang D., Dong F., Xu J., Liu X., Wu X., Pan X., Zheng Y. (2019). Enantioselective Separation and Dissipation of prothioconazole and its major metabolite prothioconazole-desthio enantiomers in tomato, cucumber, and pepper. J. Agric. Food Chem..

[15] Zhang Z., Xie Y., Ye Y., Yang Y., Hua R., Wu X. (2022). Toxifcation metabolism and treatment strategy of the chiral triazole fungicide prothioconazole in water. J. Hazard. Mater..

[16] An X., Liu X., Jiang J., Wang F., Lv L., Li G., Wu S., Zhao X. (2022). Acute and chronic toxicity of prothioconazole and its metabolite prothioconazole-desthio to Daphnia Magna. Environ. Sci. Pollut. Res..

[17] Sun Y., Cao Y., Tong L., Tao F., Wang X., Wu H., Wang M. (2020). Exposure to prothioconazole induces developmental toxicity and cardiovascular effects on zebrafish embryo. Chemosphere.

[18] Baruah P., Chaurasia N. (2020). Ecotoxicological effects of alpha-cypermethrin on freshwater alga *Chlorella* sp: Growth inhibition and oxidative stress studies. Environ. Toxicol. Pharmacol..

[19] Deng Y., Zhang W., Qin Y., Liu R., Zhang L., Wang Z., Diao J. (2019). Stereoselective toxicity of metconazole to the antioxidant defenses and the photosynthesis system of *Chlorella pyrenoidosa*. Aquat. Toxicol..

[20] Li L., Huang P., Li J. (2021). Enantioselective effects of the fungicide metconazole on photosynthetic activity in *Microcystis flosaquae*. Ecotoxicol. Environ. Saf..

[21] Liu C., Liu S., Diao J. (2019). Enantioselective growth inhibition of the green algae (*Chlorella vulgaris*) induced by two paclobutrazol enantiomers. Environ. Pollut..

[22] Zhang W., Cheng C., Chen L., Di S., Liu C., Diao J., Zhou Z. (2016). Enantioselective toxic effects of cyproconazole enantiomers against *Chlorella pyrenoidosa*. Chemosphere.

[23] Nong Q., Liu Y., Qin L., Liu M., Mo L., Liang Y., Zeng H. (2021). Toxic mechanism of three azole fungicides and their mixture to green alga *Chlorella pyrenoidosa*. Chemosphere.

[24] Huang L., Lu D., Diao J., Zhou Z. (2012). Enantioselective toxic effects and biodegradation of benalaxyl in *Scenedesmus obliquus*. Chemosphere.

[25] Deng Y., Beadham I., Ren H.Y., Ji M.M., Ruan W.Q. (2020). A study into the species sensitivity of green algae towards imidazolium-based ionic liquids using flow cytometry. Ecotoxicol. Environ. Saf..

[26] Pikula K., Chaika V., Zakharenko A., Markina Z., Vedyagin A., Kuznetsov V., Gusev A., Park S., Golokhvast K. (2020). Comparison of the level and mechanisms of toxicity of carbon nanotubes, carbon nanofibers, and silicon nanotubes in bioassay with four marine microalgae. Nanomaterials.

[27] Zheng S., Zhou Q., Chen C., Yang F., Cai Z., Li D., Geng Q., Feng Y., Wang H. (2019). Role of extracellular polymeric substances on the behavior and toxicity of silver nanoparticles and ions to green algae *Chlorella vulgaris*. Sci. Total Environ..

[28] Déniel M., Lagarde F., Caruso A., Errien N. (2020). Infrared spectroscopy as a tool to monitor interactions between nanoplastics and microalgae. Anal. Bioanal. Chem..

[29] Xin X., Huang G., Liu X., An C., Yao Y., Weger H., Chen X. (2017). Molecular toxicity of triclosan and carbamazepine to green algae Chlorococcum sp.: A single cell view using synchrotron-based Fourier transform infrared spectromicroscopy. Environ. Pollut..

[30] Dao L., Beardall J., Heraud P. (2017). Characterisation of Pb-induced changes and prediction of Pb exposure in microalgae using infrared spectroscopy. Aquat. Toxicol..

[31] Hadiyanto H., Khoironi A., Dianratri I., Suherman S., Muhammad F., Vaidyanathan S. (2021). Interactions between polyethylene and polypropylene microplastics and *Spirulina* sp. microalgae in aquatic systems. Heliyon.

[32] Xi J., Shao J., Wang Y., Wang X., Yang H., Zhang X., Xiong D. (2019). Acute toxicity of triflumizole to freshwater green algae *Chlorella vulgaris*. Pestic. Biochem. Physiol..

[33] Xiao X., Han Z., Chen Y., Liang X., Li H., Qian Y. (2011). Optimization of FDA–PI method using flow cytometry to measure metabolic activity of the cyanobacteria, *Microcystis Aeruginosa*. Phys. Chem. Earth.

[34] Strotmann U., Thouand G., Pagga U., Gartiser S., Heipieper H. (2023). Toward the future of OECD/ISO biodegradability testing-new approaches and developments. Appl. Microbiol. Biotechnol..

[35] Strotmann U., Pastor Flores D., Konrad O., Gendig C. (2020). New developments in bacterial toxicity testing: Improvement of the respiration inhibition test and the luminescent bacteria test. Processes.

[36] Hund-Rinke K., Schlinkert R., Schlich K. (2022). Testing particles using the algal growth inhibition test (OECD 201): The suitability of in vivo chlorophyll fluorescence measurements. Environ. Sci. Eur..

[37] Bradford M.M. (1976). A rapid and sensitive method for the quantitation of microgram quantities of protein utilizing the principle of protein-dye binding. Anal. Biochem..

[38] Hazeem L.J., Yesilay G., Bououdina M., Perna S., Cetin D., Suludere Z., Barras A., Boukherroub R. (2020). Investigation of the toxic effects of different polystyrene micro-and nanoplastics on microalgae *Chlorella vulgaris* by analysis of cell viability, pigment content, oxidative stress and ultrastructural changes. Mar. Pollut. Bull..

